# The Effects of Nano-SiO_2_ and Nano-TiO_2_ Addition on the Durability and Deterioration of Concrete Subject to Freezing and Thawing Cycles

**DOI:** 10.3390/ma12213608

**Published:** 2019-11-03

**Authors:** Fang Liu, Tonghuan Zhang, Tao Luo, Mengzhen Zhou, Weiwei Ma, Kunkun Zhang

**Affiliations:** Shaanxi Key Laboratory of Safety and Durability of Concrete Structures, Xijing University, Xi’an 710123, China

**Keywords:** nano-particles, concrete, freezing and thawing cycles, internal damage analysis, industrial CT scanning technology

## Abstract

The objective of this manuscript is to study the effects of nano-particle addition on the durability and internal deterioration of concrete subject to freezing and thawing cycles (FTCs). Fifteen nm of SiO_2_, 30 nm of SiO_2_, and 30 nm of TiO_2_ were added to concrete to prepare specimens with different contents. All the specimens were subjected to FTCs from 0 to 75. The mass of each specimen was measured once the FTCs reached 25, 50, and 75. Then the freezing and thawing resistance of the concrete was evaluated by computing the mass loss ratio. The pore fluid size distribution of the concrete was detected using nuclear magnetic resonance (NMR). The deterioration of the concrete subjected to FTCs was detected by industrial computed tomography (CT). The effect of different nano-particle sizes, different contents of nano-particles, and different types of nano-particles on the freezing and thawing resistance, the pore size, distribution, and the deterioration of the concrete were analyzed. The effects of FTCs on the pore size distribution and the internal deterioration of concrete were also studied. Compared to 30 nm-Nono-SiO_2_ (NS), 15 nm-NS had a better effect in improving the internal structure for concrete, and 30 nm-Nano-TiO_2_ (NT) also had a better effect in preventing pore and crack expansion.

## 1. Introduction

In cold areas of northeast and northwest China, long-term exposure to harsh environments results in various types of durability problems for concrete buildings [1]. The most common problems are freezing and thawing destruction, sulfate erosion, and osmosis damage, of which, freezing and thawing damage is the most harmful problem. Therefore, a study on how to improve the frost resistance of concrete is of important practical significance.

Nano-materials are ultra-fine materials with particle sizes of 1 to 100 nm, with a small particle size, large surface area and high surface energy with dimensional, surface, and volume effects. Taking advantages of nano-materials can have a significant impact on the properties of traditional materials [2,3], and can also lead to improvements in the durability of mortar and concrete [4,5,6]. In recent years, many nano-materials have been proven to show great performance in the improvement of the properties of concrete, such as nano-SiO_2_ [7,8,9,10,11,12,13,14], nano-TiO_2_ [15,16,17,18,19], nano-rice husk ash [6], nano-metakaolin [20], nano-Fe_2_O_3_ [21], nano-Al_2_O_3_ [21,22,23], carbon nano-tubes (CNTs) [24,25,26], and carbon nano-fibres (CNFs) [27,28,29,30].

Incorporation of nano-materials into concrete has been proven as an acceptable solution for enhancing the frost resistance of concrete. The influence of powder and aqueous nano-SiO_2_ (NS) on the freeze and thaw resistance of self-compacting concrete was studied by Quercia et al. [31], and the obtained results demonstrated that nano-silica efficiently used in self-compacting concrete (SCC) can improve its mechanical properties and durability. The effects of nano-SiO_2_ and nano-Al_2_O_3_ on the frost resistance of normal concrete were studied by Behfarnia and Salemi [22]. The results showed that nano-particles can improve the frost resistance of concrete and result in a more compact microstructure, and that the frost resistance of concrete containing nano-Al_2_O_3_ is better than that containing the same amount of nano-SiO_2_. Salemi and Behfarnia [32] studied the effect of nano-sillica and nano-alumina on the frost resistance of fiber-reinforced concrete; their results showed that 5% nano-sillica and 3% nano-alumina can improve the frost resistance of concrete as much as 83% and 81%, respectively.

In order to evaluate the effects of nano-particle addition on the durability and internal deterioration of concrete subjected to freezing and thawing cycles (FTCs), detailed experimental aspects will be presented in the subsequent sections. 15 nm/30 nm-SiO_2_ and 30 nm-TiO_2_ were added to concrete to prepare specimens with different contents, which are described in Section 2. The experimental procedures included freezing and thawing tests, nuclear magnetic resonance (NMR) detection, and industrial computed tomography (ICT) detection and are presented in Section 3. The effects of different nano-particle sizes, different contents of nano-particles, and different types of nano-particles on the freezing and thawing resistance, the pore size distribution, and the internal deterioration of concrete are analyzed in Section 4. The major conclusions are summarized in Section 5.

## 2. Specimen Preparation

### 2.1. Materials for Preparing Concrete

The cement used in this manuscript was ordinary P.O.42.5R Portland cement. The physical properties of Portland cement and its chemical compositions are detailed in Table 1 and Table 2, respectively.

Two types of nano-particles were used in this work. They were nano-SiO_2_ (NS), with particle sizes of 15 nm and 30 nm, and nano-TiO_2_ (NT) with a particle size of 30 nm, produced by Shanghai Maikun Chemical Co., Ltd. The appearance of nano-particles is a white powder of a small size, which is multi-microporous. The properties of nano-particles are shown in Table 3.

River sand was used as a fine aggregate with a fineness module of 2.8. Gravel with a diameter of 4.75–22.5 mm and a density of 2835 kg/m^3^ was used as a coarse aggregate. The plasticizer used in the experiment was a polycarboxylic acid superplasticizer produced by Shandong Yousuo Chemical Technology Co., Ltd., and its physical properties are shown in Table 4.

### 2.2. Mix Proportions of Specimens

The mix proportions of the specimens used in this manuscript are shown in Table 5.

All nano-particles were doped internally and the mass fraction was taken as a variable in this test. Among them, 15 nm-NS replaced 0.2%, 0.4%, 0.6%, 0.8%, 1%, 1.5%, and 2% of the cement, also, 30 nm-NS and 30 nm-NT replaced 0.2%, 0.4%, 0.6% and 0.8% of the cement. According to the principle of the orthogonal test, 15 mix proportions were used in this manuscript. All the specimens were subjected to four different amounts of FTC (0, 25, 50, and 75), therefore, 60 groups of specimens were needed, and three samples made a group of 180 total prepared samples.

### 2.3. Specimen Preparation

Application of nano-particles to the cement composites can cause nanomaterial agglomeration inside the cement matrix, which is the key problem related to the practical use of nano-particles in cement composites and has been discussed widely [33,34,35]. For the nano-particles used in this study, 15 nm-NS is hydrophobic and 30 nm-NS/NT is hydrophilic. To cast the concrete containing nano-particles, two mixing methods were used.

- Method 1 (for 15 nm-NS mixed with cement-based materials): (a) NS particles were mixed with river sand and stirred in a mixer at medium speed for 120 min; (b) cement and coarse aggregate were added to the mix; (c) water and superplasticizer were added to the mixture and stirred at a low speed.
- Method 2 (for 30 nm-NS/NT mixed with cement-based materials): (a) NS/NT particles were mixed with water and stirred in a mixer at a high speed for 60 min to form a suspension; (b) cement and fine aggregates were added to the mix at a medium speed; (c) coarse aggregates were added to the mixture and stirred at a low speed.

Finally, the mixture was poured into a cube mold with a size of 100 mm × 100 mm × 100 mm, and an external vibrator was used to facilitate compaction and reduce the number of air bubbles. The specimens were demolded after 24 h and then cured in a standard curing chamber for 28 days. After curing, the specimens were cored and polished.

## 3. Experiment

### 3.1. Fast Freezing and Thawing Test

**Figure 1:**
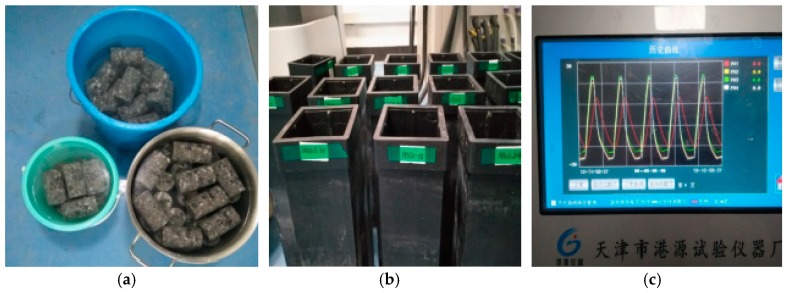
The procedures of freezing and thawing cycles test. (**a**) Test simple soaking; (**b**) Sample box marking; (**c**) Temperature of freeze thaw setup.

The specimens were subjected to cycles of freezing and thawing in an automatic fast freezing and thawing machine that can apply freezing cycles at −18 °C and thawing cycles at 5 °C, both in water. The loss of mass of specimens was measured in certain cycles (25, 50, and 75). The procedures of the freezing and thawing cycle tests is shown in Figure 1. The specimens were cylindrical with a size of Φ50 mm × 90 mm.

The mass loss rate of the concrete specimens is calculated as follows:

(1) $$\Delta W_{Ni}=\frac{W_{\mathrm{Oi}}-W_{Ni}}{W_{\mathrm{Oi}}}\times 100\%$$

where, $W_{\mathrm{Oi}}$ and $W_{Ni}$ are the initial mass before the freezing and thawing tests and the mass after N cycles of the ith specimen, respectively. $\Delta W_{Ni}$ is the mass loss rate of ith specimen after the N cycles of freezing and thawing (%).

(2) $$\Delta W_{N}=\frac{\sum_{i=1}^{3}\Delta W_{Ni}}{3}$$

where, $\Delta W_{N}$ is the average arithmetic value of mass loss rate in a group of three specimens (%).

### 3.2. Tests by Nuclear Magnetic Resonance (NMR) 

**Figure 2:**
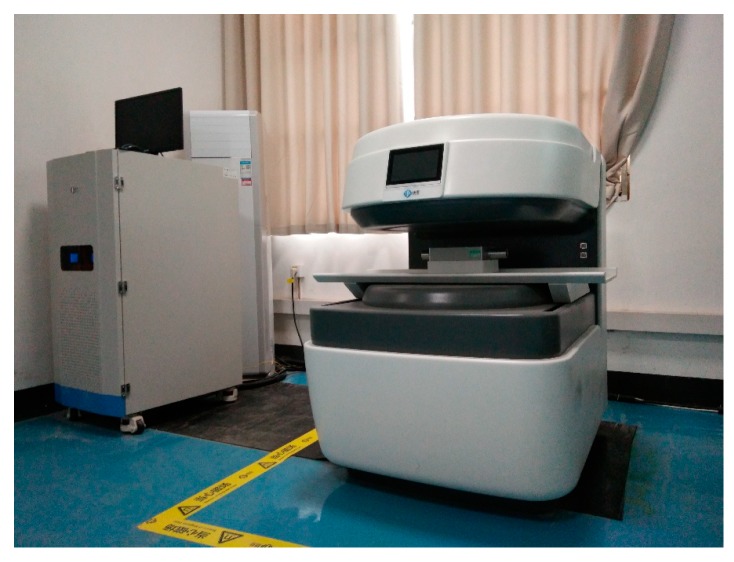
The MacroMR12-150H-I instrument.

A MacroMR12-150H-I instrument was used in this study (as shown in Figure 2). The magnetic field was 0.3 T with a resonance frequency of 50–60 Hz.

The NMR test uses the superposition signal of pore water hydrogen ions to test the Carr-Purcell-Meiboom-Gill (CPMG) pulse sequence. The attenuation signal intensity was collected and fitted by continuous curve iteration to obtain several sets of attenuation intensity curve constants, i.e., the main index T2 spectrum. If the pores in the specimen are completely filled with water, the water content measured by NMR is approximately equal to the porosity. The transverse relaxation time of the T2 spectrum is proportional to the porosity in the medium.

The reciprocal of the complete transverse relaxation rate can be expressed as shown in equation (3):

(3) $$\frac{1}{T_{2}}=\frac{1}{T_{2f}}+\frac{1}{T_{2spread}}+\frac{1}{T_{2surface}}$$

where, $\frac{1}{T_{2spread}}$ and $\frac{1}{T_{2surface}}$ are the fluid relaxation time in porous media due to magnetic field diffusion and surface relaxation, respectively. They can be calculated by Equations (4) and (5):

(4) $$\frac{1}{T_{2spread}}=\frac{D{\left(\gamma GT_{E}\right)}^{2}}{12}$$

(5) $$\frac{1}{T_{2surface}}=\rho \left(\frac{S}{V}\right)$$

where, $T_{2f}$ and $T_{E}$ are the time of fluid free relaxation and the reflected echo, respectively, ms; *D* is the diffusion coefficient, *G* is the magnetic field gradient, *G* s/cm; *γ* is the spin magnetic ratio, rad/(S·T). *S* and *V* represent the surface area and volume of the pores, respectively, ρ is the transverse surface relaxation strength of the medium (μm/s), which is 10 in this manuscript.

For porous media materials [14], such as cement-based composites, the first two items of Equation (3) can be simplified as follows, under the condition that G is taken as zero:

(6) $$\frac{1}{T_{2}}=\rho \left(\frac{S}{V}\right).$$

If the pore shape in the concrete specimen is assumed to be spherical, then:

(7) $$\frac{S}{V}=\frac{\mu }{r_{c}}.$$

The relationship between aperture and T2 spectrum can be obtained by combining Equation (7) and Equation (6):

(8) $$r_{c}=\mu T_{2}$$

where, $r_{c}$ is the aperture radius of the medium and *μ* is the empirical adjustment coefficient.

### 3.3. Industrial CT Scanning and Imaging Test

**Figure 3:**
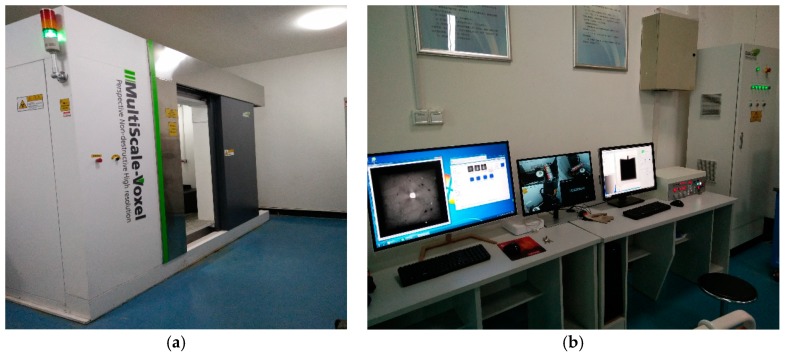
The ICT Equipment. (**a**) The ICT Scanning Equipment; (**b**) The ICT Operating Equipment.

The Multiscale Voxel series industrial CT (ICT) used in this study was the MS-Voxel 450, as shown in Figure 3. The CT uses a small focus and high power X-Ray ray source, a high sensitivity detector, and a high precision motion control system, which can ensure high spatial resolution and accurate positioning accuracy in detection.

**Figure 4:**
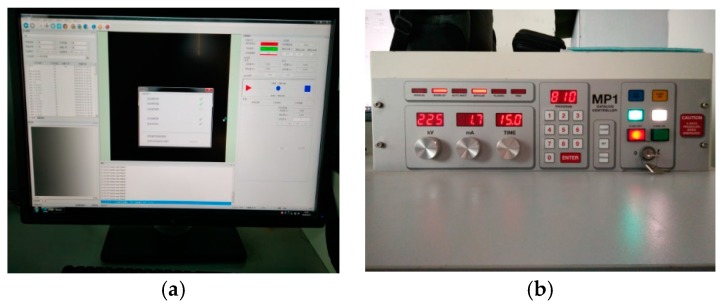
The Preparations for ICT. (**a**) Debugging Setup Interface; (**b**) ICT Radiation Source Preheating.

**Figure 5:**
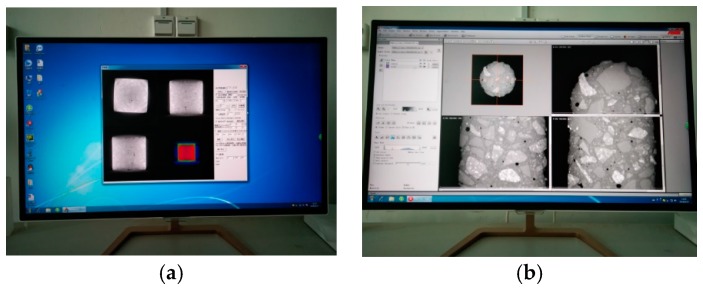
Preliminary Data Processing. (**a**) Data Pre-processing Interface; (**b**) Avizo Data Post-Processing Interface.

The specimen was placed on the sample table, which can rotate 360 degrees during testing, and then the position of the sample table was adjusted. The current and voltage were set, and the scanning back was corrected before CT testing, as shown in Figure 4. After that, the detector received an attenuation signal after the X-Ray penetrated the sample at a certain angle. Then, the collected signal was pre-processed by the pre-processing program to obtain the preliminary sectional projection image, as shown in Figure 5a. Finally, the sample was rebuilt by Avizo software, as shown in Figure 5b.

## 4. Results and Discussion

### 4.1. Freeze-Thaw Durability

Table 6 shows the mass loss rate of the specimens after they were subjected to certain freezing and thawing cycles. With an increase of the freezing and thawing cycles, the mass loss rate of the specimens increases gradually, which indicates the gradually falling off process of cement paste with freezing and thawing cycles. The increasing speed of mass loss rate decreases with the increase of the freezing and thawing cycles.

For concrete specimens with an addition of different sizes of nano-particles, the mass loss rate of the 30 nm-NS specimen is larger than that of the 15 nm-NS specimen, which indicates that smaller nano-particles of NS are more effective in improving the freezing and thawing resistance of concrete. Compared with the 30 nm-NS added concrete specimen, the mass loss rate of the 30 nm-NT added concrete specimen is generally larger, which indicates that NT is not as good at resisting mass loss when subjected to freezing and thawing. When the dosage is 0.6% (15 nm NS), the mass loss rate under different freezing-thawing cycles (25, 50, and 75) is the lowest.

### 4.2. T2 Spectrum and Pore Fluid Size Distribution Tested by NMR

The evolution of fluid distribution in pore space under different freezing and thawing cycles can be transformed and judged by the T2 spectrum. To some extent, the distribution of pore fluid can reflect the pore distribution.

#### 4.2.1. Comparison of 15 nm-NS and 30 nm-NS modified concrete

(1) Comparative analysis under different freezing and thawing cycles (FTCs).

**Figure 6:**
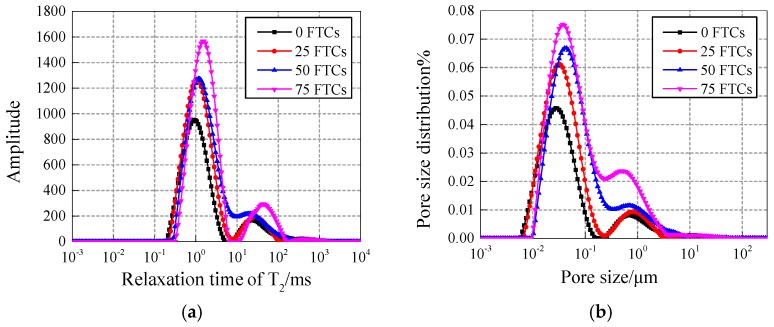
(**a**) T_2_ spectrum and (**b**) pore fluid size distribution of the 15 nm-NS specimens.

**Figure 7:**
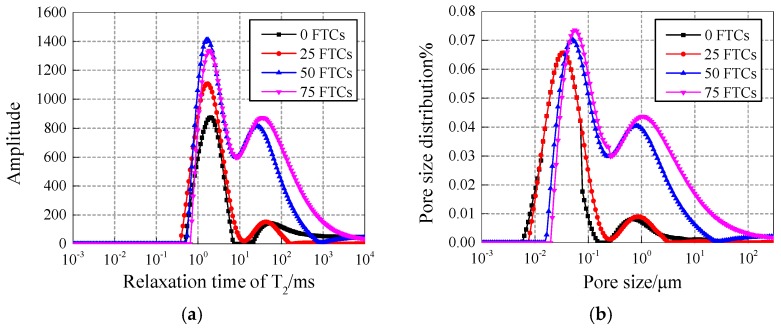
(**a**) T_2_ spectrum and (**b**) pore fluid size distribution of 30 nm-NS specimens.

Figure 6 and Figure 7 show the comparison of the T2 spectrum and pore fluid size distribution with intermediate content of 0.4% under different freezing and thawing cycles.

The curves of the 15 nm-NS and 30 nm-NS specimens show the trend of increasing peak point and right pendulum with the increase of freezing and thawing cycles, which indicates that the number, size, and volume of the pores in the specimen are increasing continuously.

(2) Analysis of the effect of different sizes of NS on the pore size distribution under different freezing and thawing cycles.

**Figure 8:**
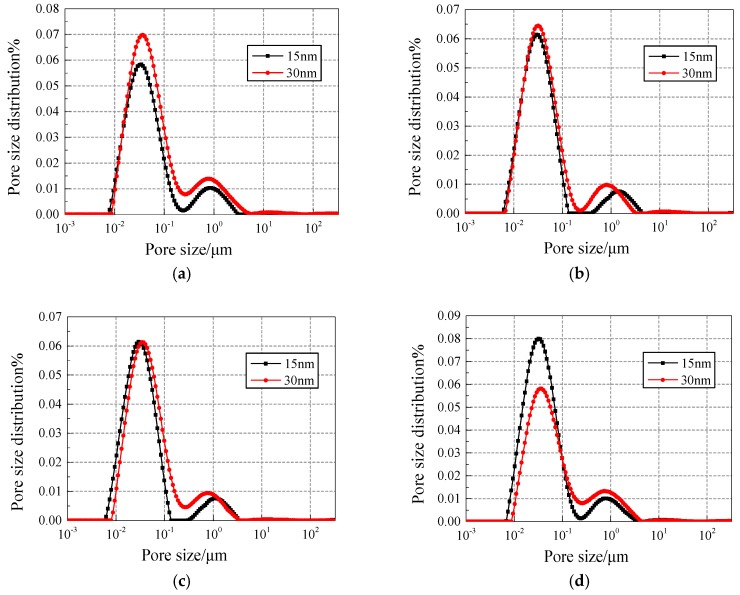
Pore fluid size distribution of NS specimens with different contents under 25 freezing and thawing cycles. (**a**) Content of 0.2%; (**b**) Content of 0.4%; (**c**) Content of 0.6%; (**d**) Content of 0.8%.

**Figure 9:**
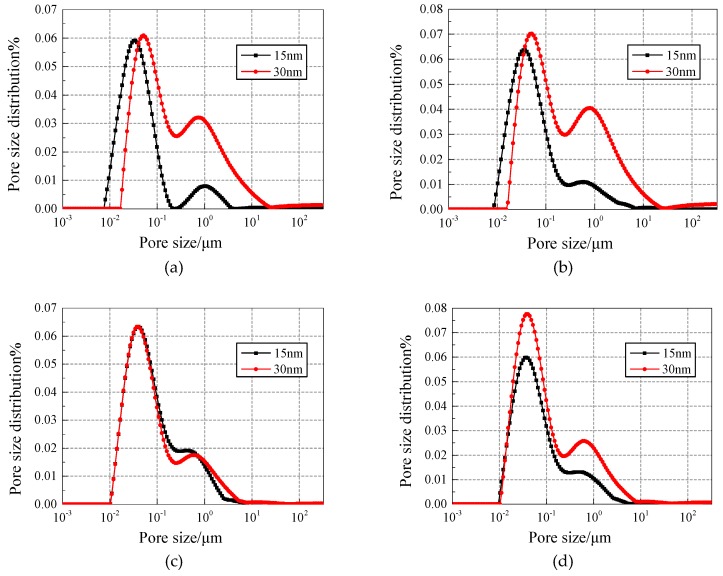
Pore fluid size distribution of NS specimens with different contents under 50 freezing and thawing cycles. (**a**) Content of 0.2%; (**b**) Content of 0.4%; (**c**) Content of 0.6%; (**d**) Content of 0.8%.

Figure 8 and Figure 9 show the pore fluid size distribution of NS specimens with different contents under 25 and 50 freezing and thawing cycles, respectively. Compared with the 15 nm-NS specimen, the 30 nm-NS specimen has more large pores in general, which explains why the 30 nm-NS specimen has lower freezing and thawing resistance. 

In Figure 9, the number of pores with a size range of 0.1–1 μm and 1–10 μm increased significantly compared with Figure 8, which explains why more freezing and thawing cycles cause more damage to concrete. 

#### 4.2.2. Comparison of NS and NT modified concrete

(1) Comparative analysis under different freezing and thawing cycles. 

**Figure 10:**
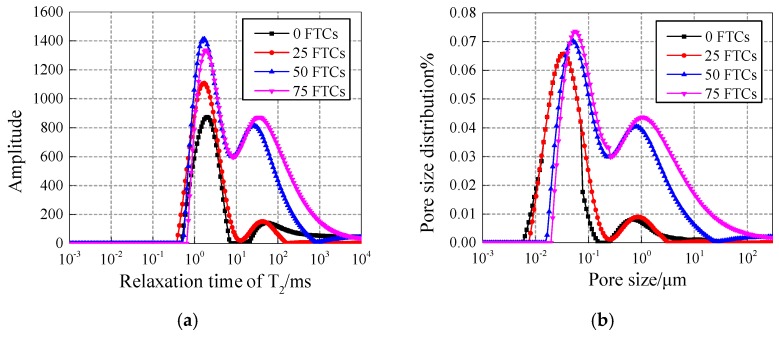
(**a**) T_2_ spectrum and (**b**) pore fluid size distribution of 30 nm-NS added concrete.

**Figure 11:**
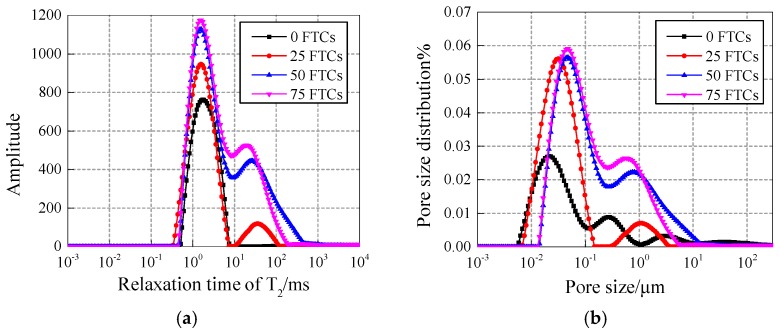
(**a**) T_2_ spectrum and (**b**) pore fluid size distribution of 30 nm-NT added concrete.

Figure 10 and Figure 11 show the T2 spectrum and pore fluid distribution of 30 nm-NS and 30 nm-NT added concrete under freezing and thawing cycles at content of 0.4%, respectively. For NS added concrete (shown in Figure 10), with the increase of the freezing and thawing cycles, both the first peak and second peak of the pore size distribution curves are mainly moving up in a vertical direction, and going right in a horizontal direction, which means that both the size of the pores and the number of larger pores increase significantly with the increase of the freezing and thawing cycles and explains why more freezing and thawing cycles cause more damage to NS added concrete. A similar conclusion can be drawn for NT added concrete from Figure 11.

(2) Direct comparison of NS and NT added concrete with different contents of nano-particle addition. 

**Figure 12:**
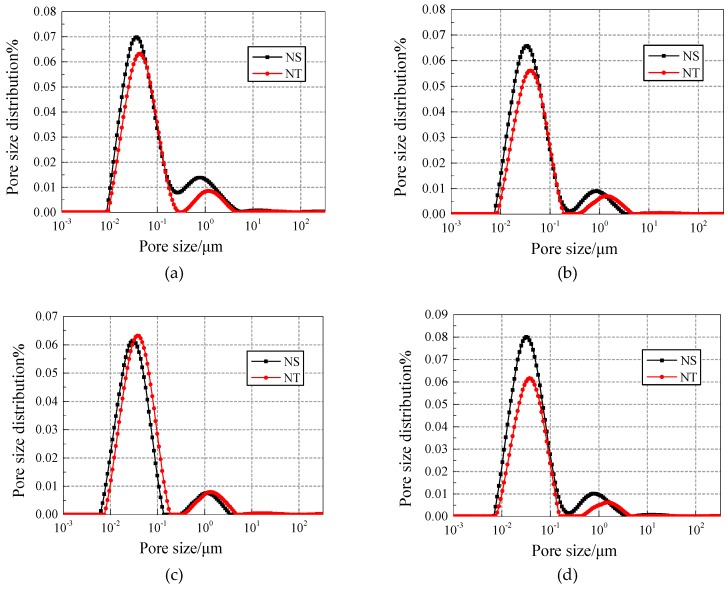
Comparison of pore fluid size distribution of NS/NT added concrete under 25 freezing and thawing cycles. (**a**) Content of 0.2%; (**b**) Content of 0.4%; (**c**) Content of 0.6%; (**d**) Content of 0.8%.

**Figure 13:**
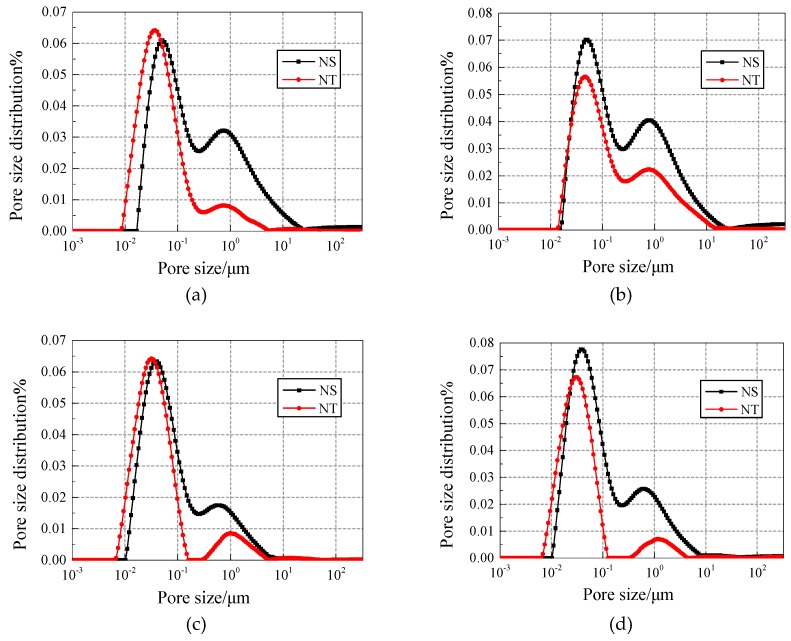
Comparison of pore fluid size distribution of NS/NT added concrete under 50 freezing and thawing cycles. (**a**) Content of 0.2%; (**b**) Content of 0.4%; (**c**) Content of 0.6%; (**d**) Content of 0.8%.

Figure 12 and Figure 13 show the pore fluid size distribution of different contents of nano-particle addition under 25 and 50 freezing and thawing cycles, respectively. Under 25 freezing and thawing cycles, in all contents of addition, both the first peak and the second peak of the pore size distribution curves for NT added concrete is on the right side of NS added concrete, but the values of the first peak and the second peak for NS added concrete are mostly larger than the NT added concrete. 

Under 50 freezing and thawing cycles, in all contents of addition, the value of the second peak of the pore size distribution curves for NT added concrete is smaller than the NS added concrete, which means NT added concrete has less harmful pores and cracks and hence has better freezing and thawing resistance. 

### 4.3. Damage Evolution Tested by ICT

In order to study the damage to the internal structure and crack evolution of nano-particle added concrete, specimens subjected to 25, 50, and 75 cycles of freezing and thawing were scanned by CT and analyzed by post-processing. 

(1) Damage evolution of nano-particle added concrete in two-dimensions.

**Figure 14:**
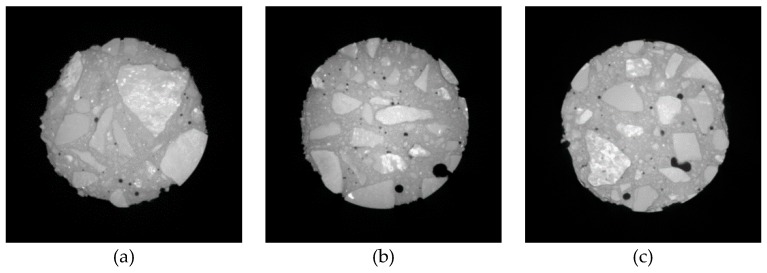

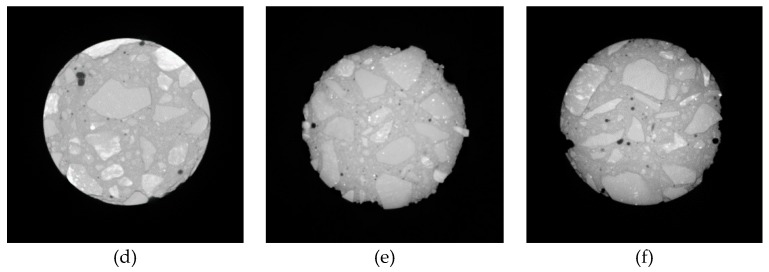
CT scanning images of different nano-particle added concrete subjected to 25 FTCs. (**a**) 15 nm-NS (0.2%); (**b**) 15 nm-NS (0.6 %); (**c**) 30 nm-NS (0.2%); (**d**) 30 nm-NS (0.4%); (**e**) 30 nm-NT (0.2%); (**f**) 30 nm-NT (0.6%).

**Figure 15:**
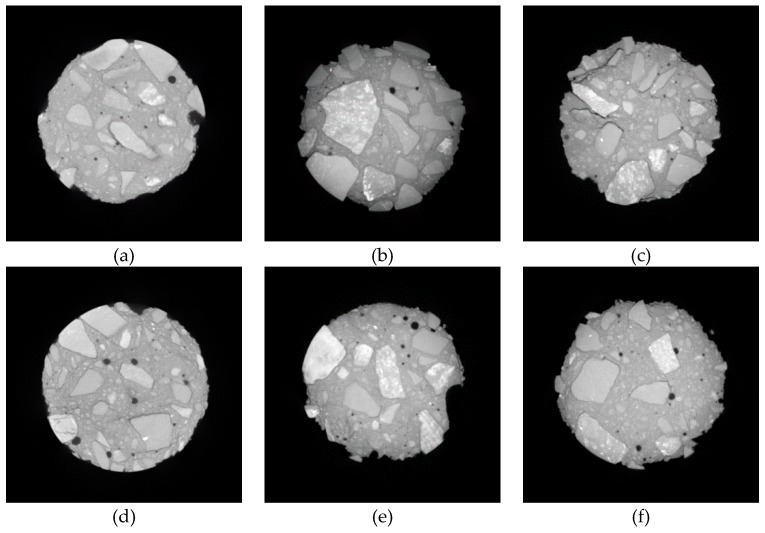
CT scanning images of different nano-particle added concrete subjected to 50 FTCs. (**a**) 15 nm-NS (0.2%); (**b**) 15 nm-NS (0.6 %); (**c**) 30 nm-NS (0.2%); (**d**) 30 nm-NS (0.4%); (**e**) 30 nm-NT (0.2%); (**f**) 30 nm-NT (0.6%).

**Figure 16:**
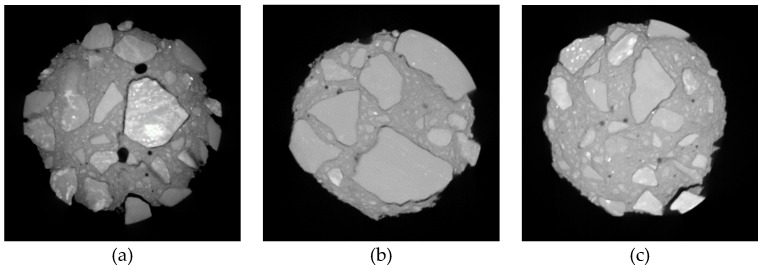

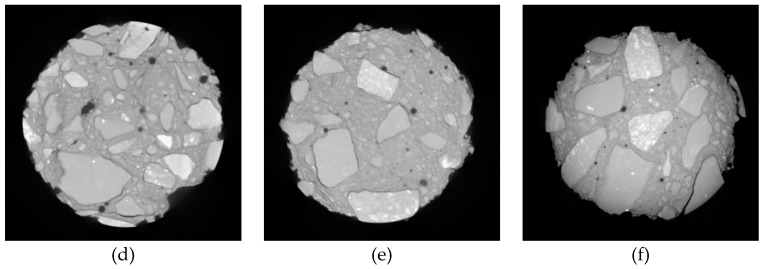
CT scanning images of different nano-particle added concrete subjected to 75 FTCs. (**a**) 15 nm-NS (0.2%); (**b**) 15 nm-NS (0.6 %); (**c**) 30 nm-NS (0.2%); (**d**) 30 nm-NS (0.4%); (**e**) 30 nm-NT (0.2%); (**f**) 30 nm-NT (0.6%).

Figure 14, Figure 15 and Figure 16 show the CT scanning images of different nano-particle added concrete subjected to 25, 50, and 75 freezing and thawing cycles (FTCs), respectively.

From Figure 14, after 25 freezing and thawing cycles, there is no significant damage in nano-particle added concrete neither for different types of nano-particles nor for different nano-particles sizes. Only slight peeling of the cement paste happens at the boundaries of the concrete specimens. From Figure 15, after 50 freezing and thawing cycles, compared with the case of 25 FTCs, the external coarse aggregate begins to fall off, and the boundaries of the specimens become more irregular. Figure 16 shows the case of 75 FTCs; the damage caused by the FTCs gradually invades the inside of the concrete specimen, the phenomenon of the peeling of the external cement paste and coarse aggregate is more serious, the boundaries of the specimens become more irregular, and expansion of cracks can be clearly seen in some specimens along the interface between the aggregate and the cement mortar.

(2) Damage evolution of nano-particle added concrete in three-dimensions.

**Figure 17:**
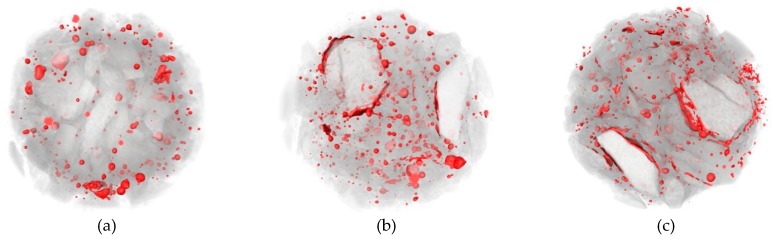
Pore distribution under different freezing and thawing cycles of 0.6% 15 nm-NS added concrete. (**a**) 25 FTCs; (**b**) 50 FTCs; (**c**) 75 FTCs.

**Figure 18:**
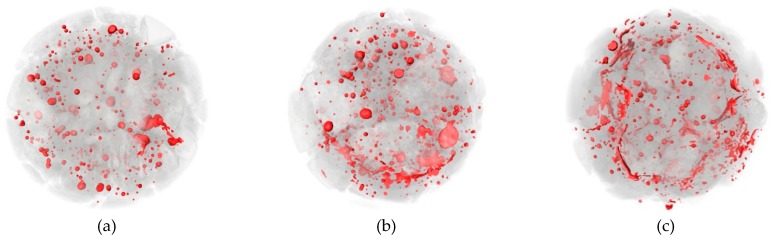
Pore distribution under different freezing and thawing cycles of 0.4% 30 nm-NS added concrete. (**a**) 25 FTCs; (**b**) 50 FTCs; (**c**) 75 FTCs.

**Figure 19:**
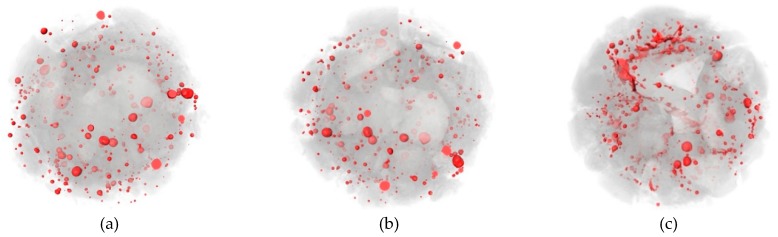
Pore distribution under different freezing and thawing cycles of 0.6% 30 nm-NT added concrete. (**a**) 25 FTCs; (**b**) 50 FTCs; (**c**) 75 FTCs.

In order to observe the pore dispersion and crack evolution within concrete under different FTCs, the pore distribution was extracted using Avizo software. Figure 17, Figure 18 and Figure 19 show the three-dimensional pore distribution under different FTCs of 0.6% 15 nm-NS, 0.4% 30 nm-NS, and 0.6% 30 nm-NT added concrete, respectively. 

From Figure 17, Figure 18 and Figure 19, after 25 cycles of freezing and thawing, it can be seen that the pores in these specimens are mainly irregular spheres. After 50 freezing and thawing cycles, pore expansion is obvious, and cracks begin to appear and expand, which is the most serious in the 0.4% 30 nm-NS added concrete. After 75 freezing and thawing cycles, many pores are connected and wrap around the coarse aggregates, forming cracks along the interface of the cement mortar and coarse aggregate, which is most obvious in the 0.4% 30 nm-NS added concrete.

Three-dimensional pore extraction and calculation were carried out using Avizo post-processing software in order to quantitatively compare the effects of nano-particle addition with different particle size and types on the internal structure of cement-based composites. Table 7 shows the total internal porosity of nano-particle added concrete under different FTCs (including pores and cracks).

From Table 7, the internal porosity in the 30 nm-NS added concrete is larger than in the 15 nm-NS added concrete, which indicates that the concrete with 15 nm-NS has better freezing and thawing resistance. At the same time, the internal porosity in the 30 nm-NT added concrete is smaller than in the 30 nm-NS added concrete, which indicates that the internal deterioration of concrete with NT is relatively light. However, there is no obvious regularity between the comparison of 15 nm-NS added concrete and 30 nm-NT added concrete. 

## 5. Conclusions

In this study, fast freezing and thawing cycle tests, nuclear magnetic resonance detection, and Industrial CT scanning tests were carried out for 15/30 nm-NS and 30 nm-NT added concrete specimens. The effects of different nano-particle sizes and types on the freezing and thawing resistance and internal structure of concrete were analyzed. Based on the experimental results and discussion in this study, the following primary conclusions can be obtained:

(1) Under freezing and thawing cycles, the mass loss rate in 15 nm-NS added concrete is smaller than in 30 nm-NS added concrete, and the number of harmful holes is also less than in the 30 nm-NS added concrete, as is the total internal porosity, which means that 15 nm-NS has a better effect in improving the internal structure for concrete and hence improves the freezing and thawing resistance.

(2) With the increase of freezing and thawing cycles, the 30 nm-NT added concrete has a smaller number of harmful pores and less total internal porosity than the 30 nm-NS added concrete. From the three-dimensional pore distribution, it can be concluded that 30 nm-NT has a better effect in preventing pore and crack expansion. 

(3) Damage evolution of nano-particle added concrete in two-dimensions and three-dimensions were discussed in this study. With the increase of freezing and thawing cycles, the damage caused by FTCs gradually invades the inside of the concrete specimen, the phenomenon of the peeling of the external cement paste and coarse aggregate becomes serious, the boundaries of the specimens become more irregular, and expansion of cracks can be clearly seen in some specimens along the interface between the aggregate and the cement mortar.

## Figures and Tables

**Table 1:** The physical properties of Portland cement.

Density (g/cm^3^)	Fineness (%)	Specific Surface Area (m^2^/g)	Stability	Setting Time (min)	
				Initial Setting	Final Setting
3.10	≤8.0	0.36	Qualified	≥60	≤300

**Table 2:** The chemical compositions of Portland cement (%).

CaO	SiO_2_	Al_2_O_3_	Fe_2_O_3_	MgO	SO_3_	Alkali	Ignition Loss
61.43	22.81	5.62	3.36	1.35	2.17	0.54	2.60

**Table 3:** The properties of nano-particles.

Item	Size (nm)	Formula Weight	Purity (%)	Ignition Loss (%)	Specific Surface Area(m^2^/g)	PH
Nano-SiO_2_ (NS)	15	60.08	99.8	0.2	250	5–7
	30		99.5	0.5	220	
Nano-TiO_2_ (NT)	30	79.88	99	1	45	6–8

**Table 4:** The physical properties of polycarboxylic acid superplasticizer.

Appearance	Hydroxyl	PH	Water Content	Solubility
Light yellow to white flakes	22–27	5.0–7.0	≤0.5	Soluble in water and other organic

**Table 5:** The mix proportions of specimens.

Specimen	W/C	C/kg	Aggregate		Nano-Particles/g			W/kg	SP/g
			S/kg	G/kg	15nm-NS	30 nm-NS slurry	30 nm-NT slurry		
NS0.2-15	0.5	8.77	14.28	26.54	17.57	0	0	4.39	0
NS0.4-15	0.5	8.75	14.28	26.54	35.14	0	0	4.39	0
NS0.6-15	0.5	8.73	14.28	26.54	52.70	0	0	4.39	0
NS0.8-15	0.5	8.71	14.28	26.54	70.27	0	0	4.39	0
NS1-15	0.5	8.70	14.28	26.54	87.84	0	0	4.39	0
NS1.5-15	0.5	8.65	14.28	26.54	131.76	0	0	4.39	0
NS2-15	0.5	8.61	14.28	26.54	175.68	0	0	4.39	0
NS0.2-30	0.5	8.77	14.28	26.54	0	105.39	0	4.30	17.56
NS0.4-30	0.5	8.75	14.28	26.54	0	210.77	0	4.22	21.95
NS0.6-30	0.5	8.73	14.28	26.54	0	316.16	0	4.13	26.34
NS0.8-30	0.5	8.71	14.28	26.54	0	421.55	0	4.04	30.73
NT0.2-30	0.5	8.77	14.28	26.54	0	0	105.39	4.30	17.56
NT0.4-30	0.5	8.75	14.28	26.54	0	0	210.77	4.22	21.95
NT0.6-30	0.5	8.73	14.28	26.54	0	0	316.16	4.13	26.34
NT0.8-30	0.5	8.71	14.28	26.54	0	0	421.55	4.04	30.73

Note: NS is nano-SiO_2_; NT is nano-TiO_2_; W/C is water binder ratio; C is cement; S is sand; G is coarse aggregate; W is water; SP is superplasticizer; 30 nm-NS slurry is the mix of 30 nm nano-SiO_2_ and water with a ratio of 1:5; 30 nm-NT slurry is the mix of 30 nm nano-TiO_2_ and water with a ratio of 1:5.

**Table 6:** The mass loss rate of specimens after certain freezing and thawing cycles (%).

Different Contents (%)	25 Cycles			50 Cycles			75 Cycles		
	15 nm-NS	30 nm-NS	30 nm-NT	15 nm-NS	30 nm-NS	30 nm-NT	15 nm-NS	30 nm-NS	30 nm-NT
0.2	1.77	1.88	1.94	3.28	3.56	3.66	4.00	4.36	4.38
0.4	1.11	1.35	1. 77	2.57	2.95	3.00	3.71	3.55	4.12
0.6	0.96	1.48	1.52	2.20	3.16	2.66	3.42	3.87	3.99
0.8	1.25	1.98	1.86	3.01	3.43	3.38	4.00	3.90	4.20
Average	1.27	1.67	1.77	2.77	3.28	3.18	3.78	3.92	4.17

**Table 7:** The internal porosity of nano-particle added concrete under different FTCs (%).

Different Content %	25 FTCs			50 FTCs			75 FTCs		
	15 nm	30 nm		15 nm	30 nm		15 nm	30 nm	
	NS	NS	NT	NS	NS	NT	NS	NS	NT
0.2	1.66	1.96	1.58	1.97	2.52	1.99	1.98	3.45	2.04
0.4	1.62	1.57	1.57	1.75	1.94	1.60	1.90	3.25	2.08
0.6	1.48	1.66	1.35	1.69	2.43	1.56	1.79	2.38	1.42
0.8	1.68	2.03	1.43	1.81	2.90	1.72	1.89	3.35	1.94
